# Achieving diagnostic excellence for infectious keratitis: A future roadmap

**DOI:** 10.3389/fmicb.2022.1020198

**Published:** 2022-10-03

**Authors:** Darren S. J. Ting, James Chodosh, Jodhbir S. Mehta

**Affiliations:** ^1^Academic Ophthalmology, School of Medicine, University of Nottingham, Nottingham, United Kingdom; ^2^Department of Ophthalmology, Queen's Medical Centre, Nottingham, United Kingdom; ^3^Department of Ophthalmology, Massachusetts Eye and Ear and Harvard Medical School, Boston, MA, United States; ^4^Department of Cornea & Refractive Surgery, Singapore National Eye Centre, Singapore, Singapore; ^5^Singapore Eye Research Institute, Singapore, Singapore

**Keywords:** corneal ulcer, corneal infection, microbial keratitis, infectious keratitis, artificial intelligence, next generating sequencing, digital technologies, diagnosis

## Introduction

Infectious keratitis (IK), also known as corneal infection, is the 5^th^ leading cause of vision impairment and blindness globally (Flaxman et al., 2017). Once recognized as a “silent epidemic,” the incidence of IK is now estimated at 2.5–799 per 100,000 population-year, with a significantly higher incidence noted in low- and middle-income countries (LMICs) (Ting et al., 2021a). It has so far resulted in ~5 million cases of blindness and/or significant vision impairment and is estimated to account for 1.5–2.0 million cases of monocular blindness per year. A recent systematic review estimated that fungal keratitis alone affects >1 million people annually, particularly in Africa and Asia, particularly China, India and Nepal (Brown et al., 2021). In addition, approximately $175 million dollars are being spent on IK annually within the healthcare systems of the United States. In view of the significant burden on the global population, healthcare system and economy, a recent international consortium has proposed the addition of IK to the list of neglected tropical diseases (NTDs), with an aim to draw global concerted and sustained efforts in tackling this important disease (Ung et al., 2019).

IK is a painful and potentially sight-threatening condition that often requires intensive medical and/or surgical interventions (Khor et al., 2018; Ting et al., 2021b). Complications such as corneal melt, perforation, endophthalmitis and complete loss of the eye may sometimes ensue despite intensive treatment (Khor et al., 2018; Cabrera-Aguas et al., 2021; Ting et al., 2021b,c). A timely and accurate diagnosis is often the key to a successful clinical outcome, though this is not always possible in clinical practice due to various barriers (Table 1). In this Opinion article, we discuss the current diagnostic modalities for IK and their limitations and highlight the potential solutions that can be offered by the recent advancement in digital innovations and clinical metagenomic next generation sequencing (NGS).

**Table 1:** Current diagnostic challenges of infectious keratitis (IK) and future potential solutions enabled by digital innovations and clinical metagenomic next generation sequencing (NGS).

**Current challenges**	**Underlying reasons**	**Potential solutions**
Delayed diagnosis	• Limited access to healthcare systems, particularly in LMICs. • Lack of clinical expertise and resources. • Poor awareness/education of the condition among the patients. • Use of traditional eye medicine delaying the presentation to healthcare professionals.	• Teleophthalmology enables real-time, synchronous, remote assessment by the clinicians, enabling a timely diagnosis. • AI-assisted tools may allow for timely and automated diagnosis with limited input from clinical experts.
Undifferentiated clinical phenotypes	• IK, either due to bacterial, fungal, parasitic and/or viral, usually presents as corneal infiltrate(s)/abscess (appearing as a white corneal opacity).	• AI-assisted tools may help distinguish the underlying causes of IK (e.g., bacterial keratitis vs. fungal keratitis).
Low culture yield	• Prior use of topical antimicrobial treatment before the diagnosis. • Low bioburden with limited infectious sample compared to systemic infection. • Poor techniques in corneal sampling and/or microbiological processing.	• NGS provides higher sensitivity and specificity results than conventional microbiological culture techniques. This is particularly useful for disease such as IK with low infectious bioburden. • Close collaboration between the ophthalmologists and the microbiologists to refine the sampling-to-processing pathway.
Long turnaround time for culture results	• The incubation period is usually 1–2 days for bacteria (or longer for some atypical bacteria) and 5–14 days for fungi.	• NGS can provide the results within 24 h (particularly with short-read, targeted amplicon sequencing). This is especially valuable for cases that are affected by atypical bacteria or fungi.
Polymicrobial infection	• The ocular surface (including cornea) is exposed to a multitude of organisms and potential risk factors (e.g., trauma, CL, etc.).	• NGS can sequence all types of infection in parallel. • NGS can also facilitate the examination of antimicrobial resistance and virulence genes (usually achieved by long-read, whole genome sequencing).

LMICs, Low- and middle-income countries; AI, Artificial intelligence; CL, Contact lens.

## Current diagnostic approach and limitations

IK is primarily diagnosed on clinical grounds with support of microbiological findings. It is characterized by corneal ulceration, inflammation and/or infiltrate (an abscess manifesting as a white corneal opacity). A wide range of pathogens, including bacteria, fungi, parasites, and viruses, are capable of causing IK. However, the clinical features are often poorly differentiated, especially in patients with late presentation (common in LMIC populations), and may be complicated by the presence of polymicrobial infections (occurring in 2–15%) (Ting et al., 2019a, 2021a; Khoo et al., 2020). Several studies have highlighted the inability of corneal experts to accurately determine the underlying cause of IK (based on clinical photographs) in >50% cases, highlighting the diagnostic challenges based on clinical presentation alone (Redd et al., 2022a).

Corneal sampling for microbiological investigations such as microscopy, culture and sensitivity testing is the current “gold standard” in clinical practice to help determine the underlying causative pathogens and guide the choice of antimicrobial treatment. Nonetheless, due to a considerably low bioburden in IK (as compared to systemic infections), the culture yield is hindered by invariably low sensitivity (30–50%) and slow turnaround time for positive results (e.g., the incubation time may take up to 2 weeks for certain pathogens). A negative culture result can lead to significant therapeutic challenges, particularly in cases with poor initial treatment response to broad-spectrum antimicrobial therapy, as clinicians are often pressured to subject the affected patients to polypharmacy (i.e., concurrent use of different antimicrobials) to ensure the maximal coverage of possible causative organisms (a “blanket approach”). This can in turn lead to undesirable dose-dependent ocular toxicity, impede corneal healing and exacerbate corneal ulceration/melt, culminating in corneal opacity and blindness.

The limitations of the current diagnostic approaches are varied in different settings and may be more specific or relevant to certain populations/countries. For instance, in LMICs, the lack of access to healthcare services and the lower level of patient education can result in late presentation of the disease. This can lead to poorly differentiated clinical features, less accurate diagnosis, and most importantly, worse clinical outcomes. In addition, the relative lack of resources in such settings (e.g., microbiological services and facilities) limits the clinician's capacity to make the clinical diagnosis. On the other hand, patients residing in high-income countries (HICs) have better access to healthcare systems, and in these settings, improving the likelihood of a microbiologic diagnosis would be beneficial. Understanding the diagnostic limitations specific to certain regions or populations will enable a more efficient and effective implementation of future innovations for improving the diagnosis and treatment of IK.

## Potential solutions for improving diagnostic performance

### Digital innovations

In recent years, the simultaneous advancement in deep learning (DL)-based artificial intelligence (AI) techniques, computer processing power, internet-of-things, and big data analytics have unlocked the potential of digital health. Within ophthalmology, AI-assisted models have facilitated image-based automated medical diagnosis for a range of ocular diseases and assisted in clinical triage and decision making, thereby enhancing the workflow efficiency in healthcare services in both high-income countries (HICs) and LMICs (Li et al., 2020, 2021; Rampat et al., 2021; Tiwari et al., 2022). Recently, Li et al. (2020) demonstrated the potential of using a DL-based algorithm with densely annotated slit-lamp photographs to accurately diagnose and distinguish various ocular diseases, including IK, conjunctivitis, pterygium, and cataract, and to provide automated treatment recommendation. The AI model was further tested prospectively in a real-world setting and demonstrated high accuracy (>90%) in making the correct treatment recommendation, with good end-user satisfaction (including both patients and doctors).

In addition, DL-based models have demonstrated good accuracy in differentiating the underlying cause of IK, particularly between bacterial and fungal keratitis (Hung et al., 2021; Redd et al., 2022b), which is a common diagnostic dilemma in clinical practice. Redd et al. (2022b) recently evaluated five convolutional neural networks using images of culture-proven IK obtained from handheld cameras and reported a higher diagnostic accuracy of the CNN-based DL models when compared to cornea experts (area under the ROC = 0.84 vs. 0.76). Another study has also shown the ability of a CNN-based DL model in accurately distinguishing between corneal scars and IK (Tiwari et al., 2022), which serves as a useful and inexpensive system to aid the triage, assessment, initiation and cessation of antimicrobial therapy for IK in regions with limited access to eye care.

### Clinical metagenomic next generation sequencing

Polymerase chain reaction (PCR)-based molecular diagnostics have proven to be a useful diagnostic tool for various infectious diseases, including IK (Yang and Rothman, 2004). The advent of next generation sequencing (NGS), a technology that allows for rapid, massive parallel sequencing of DNA and RNA, has significantly increased the efficiency and quality of sequencing, with significantly reduced costs (Goodwin et al., 2016). It can be performed using either two approaches, namely short-read sequencing (or targeted-amplicon sequencing; TAS) or long-read sequencing (or whole-genome sequencing; WGS), depending on clinical needs. TAS allows for a more rapid sequencing with quicker yield of results, but at the expense of reduced comprehensiveness than WGS, which provides a higher resolution of genomic examination, facilitating the study of antimicrobial resistance and virulence genes, disease transmission and potential outbreaks.

So far, NGS has demonstrated promise in augmenting clinical microbiology and public health laboratory practice (Gwinn et al., 2019), and its applicability is now gradually gaining traction in the field of IK (Ung et al., 2020). Owing to its high sensitivity and specificity, studies have shown that NGS is able to yield positive results in culture-negative IK cases (where the infectious bioburden is usually low or the infection is caused by atypical organisms or fungi), with a quick turnaround time (Seitzman et al., 2019a,b). It can sequence all types of organisms, including bacteria, fungi, viruses, and parasites, an advantage that is particularly valuable for IK where polymicrobial infection is relatively common. It was also able to identify new pathogens that were previously unknown to be associated with ocular infections. Seitzman et al. (2019b) reported the use of metagenomic NGS in aiding the diagnosis of a case of *Capnocytophaga spp*.-related keratitis, where all the traditional microbiological techniques (including Gram stain, potassium hydroxide stain, and culture), corneal biopsy, and *in vivo* confocal microscopy were unrevealing. During the first 2 months of treatment (prior to the correct diagnosis), the patient progressively worsened despite being on various antibiotic and antifungal treatments. Fortunately, the initiation of the appropriate therapy (topical clindamycin 5%) successfully eradicated the infection and inflammation within 6 weeks, highlighting the clinical value of metagenomic NGS in IK. Moreover, the sensitivity of NGS is not affected by the prior use of antimicrobials, which is a common impediment to culture yield (a common issue that is observed in HICs due to widespread use of antibiotics).

## Discussion

The diagnostic modalities for IK have remained largely similar for the past few decades, with conventional microbiological technique being the most prevailing method. Digital innovations such as AI (particularly DL) and telemedicine have demonstrated their potential clinical utility in improving the rapidity, efficiency and accuracy for diagnosing IK in the recent years, though the deployment of such technology in this field still remains in its infancy. Other than diagnosing and distinguishing IK from other ocular surface diseases, potential AI research areas for IK may further include the prediction of culture positivity, clinical outcomes and complications of IK, including need for surgical interventions, based on the initial clinical presentations and photos. These digital innovations can provide a much-needed solution in LMICs where IK is most prevalent and healthcare services are constrained by physical, financial, workforce, societal, environmental and policy limitations (Mills, 2014). An ideal model of care would be an AI-assisted teleophthalmology or mHealth-based platform which allows the non-ophthalmologists (e.g., trained technicians) or affected patients to capture and upload the corneal photos remotely using either slit-lamp photography (in dedicated community facilities) or mobile device, respectively, followed by cloud-based, AI-assisted automated analysis and clinical decision making (Ting et al., 2019b).

Although NGS has demonstrated significant promise in improving the diagnosis of various types of infection, the routine use of NGS in clinical practice is hindered by its high cost (i.e., around $150–200 for each NGS per patient), though parallel sequencing of multiple samples and pathogens may improve laboratory efficiency and reduce cost. In addition, NGS can result in non-selective amplifications of the RNA / DNA of the causative organisms and normal ocular surface commensals, which will require special bioinformatic consideration and analysis (hence the need for additional resources, facilities and expertise). Another issue that has been highlighted is that NGS may produce inconsistent results to the conventional culture methods, which may complicate the clinical findings and decision making in IK (An et al., 2022).

Broad-spectrum antimicrobial therapy is currently the mainstay of treatment for IK, and studies have shown that the majority of IK pathogens are usually susceptible to the commonly used antibiotics (Ting et al., 2021d). In view of the above-highlighted limitations of NGS at its current state, we suggest that the use of NGS is best reserved for selective cases of IK where the initial treatment response was unsatisfactory and in regions where antimicrobial resistant ocular pathogens are more prevalent (e.g., the USA, China and India) (Ting et al., 2021a).

In conclusion, timely and accurate diagnosis of IK is critical in achieving a good outcome, though many barriers still exist. It is envisaged that the continual evolution of digital innovations and NGS will likely transform the diagnostic landscape of IK in the coming years. Further considerations and effort are required to improve the infrastructure, accessibility, costs and cost-effectiveness of these technologies to facilitate their implementations, particularly in LMICs.

## Author contributions

DT and JM: study design and conceptualization. DT: literature review and manuscript drafting. JC and JM: critical revision of the manuscript. DT, JC, and JM: final approval of the manuscript. All authors contributed to the article and approved the submitted version.

## Funding

DT acknowledges support from the Medical Research Council/Fight for Sight (FFS) Clinical Research Fellowship (MR/T001674/1).

## Conflict of interest

The authors declare that the research was conducted in the absence of any commercial or financial relationships that could be construed as a potential conflict of interest.

## Publisher's note

All claims expressed in this article are solely those of the authors and do not necessarily represent those of their affiliated organizations, or those of the publisher, the editors and the reviewers. Any product that may be evaluated in this article, or claim that may be made by its manufacturer, is not guaranteed or endorsed by the publisher.
